# The transcription factor *MebHLH18* in cassava functions in decreasing low temperature-induced leaf abscission to promote low-temperature tolerance

**DOI:** 10.3389/fpls.2022.1101821

**Published:** 2023-02-13

**Authors:** Wenbin Liao, Jie Cai, Haixia Xu, Yilin Wang, Yingjie Cao, Mengbin Ruan, Songbi Chen, Ming Peng

**Affiliations:** ^1^ Institute of Tropical Bioscience and Biotechnology, Chinese Academy of Tropical Agricultural Sciences, Haikou, China; ^2^ Hainan Institute for Tropical Agricultural Resources, Institute of Tropical Bioscience and Biotechnology, CATAS, Haikou, China; ^3^ Tropical Crops Genetic Resources Institute, Chinese Academy of Tropical Agricultural Sciences/Key Laboratory of Ministry of Agriculture for Germplasm Resources Conservation and Utilization of Cassava, Haikou, China

**Keywords:** transcription factor bHLH, low temperature, leaf abscission, GWAS, ROS scavengers

## Abstract

The reactive oxygen species (ROS) signal regulates stress-induced leaf abscission in cassava. The relationship between the function of the cassava transcription factor *bHLH* gene and low temperature-induced leaf abscission is still unclear. Here, we report that *MebHLH18*, a transcription factor, involved in regulating low temperature-induced leaf abscission in cassava. The expression of the *MebHLH18* gene was significantly related to low temperature-induced leaf abscission and POD level. Under low temperatures, the levels of ROS scavengers in different cassava genotypes were significantly different in the low temperature-induced leaf abscission process. Cassava gene transformation showed that *MebHLH18* overexpression significantly decreased the low temperature-induced leaf abscission rate. Simultaneously, interference expression increased the rate of leaf abscission under the same conditions. ROS analysis showed a connection between the decrease in the low temperature-induced leaf abscission rate caused by *MebHLH18* expression and the increase in antioxidant activity. A Genome-wide association studies analysis showed a relationship between the natural variation of the promoter region of *MebHLH18* and low temperature-induced leaf abscission. Furthermore, studies showed that the change in *MebHLH18* expression was caused by a single nucleotide polymorphism variation in the promoter region upstream of the gene. The high expression of *MebHLH18* led to a significant increase in POD activity. The increased POD activity decreased the accumulation of ROS at low temperatures and the rate of leaf abscission. It indicates that the natural variation in the promoter region of *MebHLH18* increases antioxidant levels under low temperatures and slows down low temperature-induced leaf abscission.

## Introduction

Cassava (*Manihot esculenta* Crantz) is an important food crop and a potential biomass energy source worldwide. It is the sixth-largest food crop in the world and is a staple food for 700 million people (Wang et al., 2014; Liao et al., 2016a; Liao et al., 2016b). Cassava originated in warm climates, with high light efficiency, starch yield, drought resistance, barren resistance, and other characteristics (Liao et al., 2016a). However, it has a low tolerance to low-temperature stress. Therefore, low temperature is an important limiting factor that restricts the transplantation of cassava to high latitude areas and the expansion of its productivity. It is necessary to mine low-temperature tolerant cassava germplasms, deeply analyze its genetic mechanisms, and then select new low-temperature tolerant cassava varieties. This exploration significantly benefits cassava production in the north. Cassava has a very obvious occipital abscission tissue that controls the abscission and growth of leaves. The mechanisms of leaf abscission and growth conversion in cassava demonstrate their strong adaptability to adversity. Under adverse conditions, cassava actively abscises a part of its leaves to adapt and avoid adverse environments. However, under the appropriate environmental conditions after the removal of adversity, cassava can flexibly grow new leaves to meet the needs of efficient photosynthesis, which is a structural characteristic of cassava different from other crops (Liao et al., 2016a). Stress-induced flexible shedding and growth transition of leaves in the occipital abscission zone of cassava have a complex mechanism. Studies have shown that many genes regulate this process, and the reactive oxygen species (ROS) signal is the key regulatory signal (Liao et al., 2016a).

Plants deal with low temperatures and other stresses using complex and diverse mechanisms. It includes physical and structural adaptations, the role of intercellular regulatory permeable substances, and the clearance of ROS by protective enzyme systems. The ROS signaling pathway has a key position in the regulatory network (Lee and Lee, 2000). ROS are by-products of metabolic processes in the mitochondria, chloroplasts, and peroxisomes. High levels of ROS damage cells by attacking the cell membranes, destroying proteins, lipids, and other cellular components, destroying defense barriers, and so on. The production and clearance of ROS in plants are in a state of dynamic balance when the growth environment is normal. Hydroxyl radicals (OH-), superoxide anion radicals (O_2_·-), and hydrogen peroxide (H_2_O_2_) are a few toxic ROS that is produced in excess during adverse conditions (Sagi and Fluhr, 2006). Peroxidase (POD), catalase (CAT), and superoxide dismutase (SOD) in the antioxidant enzyme system can effectively scavenge ROS, enhancing plants tolerance to adversity (Gechev et al., 2006). The antioxidant enzyme system regulates the stress resistance of cassava and leaf senescence (Xu et al., 2013a). During the growth and development of cassava, an effective ROS scavenging mechanism can delay the senescence of stems and leaves, maintain the leaves for a certain level of photosynthesis, and thus affect the starch accumulation and final yield of cassava. However, shoot photosynthesis must be maintained within a certain range, as too much or too little does not promote starch accumulation (Luo et al., 2007). The ability of cassava to scavenge ROS was significantly improved by the simultaneous overexpression of two ROS-scavenging enzyme genes, *MeCu/ZnSOD* and *MeCAT1*. It was superior to the wild-type control in terms of green retention of leaves, anti-aging, post-harvest rot resistance of root tubers, and resistance to adverse conditions. However, there was no significant difference between the final root tuber yield and the control (Xu et al., 2013a; Xu et al., 2013b). Liao et al. confirmed that excessive ROS accumulation could start cassava leaf abscission process so that the abscission can be completed before its life cycle is completed (Liao et al., 2016a; Liao et al., 2016b).

Plant *bHLH* is an important transcription factor gene that widely regulates low temperature and ROS response. *CsbHLH18*, a sweet orange transcription factor, regulates the cold tolerance and homeostasis of plant mainly by regulating the production of ROS using antioxidant genes (Geng and Liu, 2018). In transgenic *Arabidopsis*, overexpression of the *FtbHLH2* gene in *Fagopyrum tataricum* improves low-temperature tolerance, and this enhancement of low-temperature tolerance is related to ROS (Yao et al., 2018). Overexpression of the *HLH* transcription factor *RmICE1* improves the ability to tolerate low temperatures by regulating the level of ROS and activating the expression of stress response genes in *Rosa roxburghii* (Luo et al., 2020). Other plants, including the *Cryptomeria fortunei*, the cherry (*Prunus avium L.*), and the longan (*Dimocarpus longan*), have been reported to use bHLH transcription factors in response to low temperatures and ROS (Yang et al., 2019; Shen et al., 2021; Zhang et al., 2021).

Materials with various genotypes are important resources for studying the mechanisms of overcoming adversity (Basu et al., 2016). The different ROS scavenging mechanisms between genotypes may explain the variation in stress resistance. When subjected to stress, stress-sensitive plants have high ROS levels and low enzyme activity to eliminate them, ultimately leading to serious cell membrane damage. The ROS level of stress-resistant plants is lower than that of sensitive plants, and the enzyme activity of scavenging ROS is increased, which makes the degradation of the growth phenotype of stress-resistant genotypes lower under stress (Siddiqui et al., 2015; Ren et al., 2016). Under adverse conditions, the stress-resistant materials have high antioxidant activity, low ROS content, and lipid peroxidation, as well as higher growth potential, yield, and yield components. In contrast, stress-sensitive materials may have lower yield potential under adverse conditions due to defects in the antioxidant system of vegetative and reproductive organs (Singh et al., 2012). Here, we report that the transcription factor *MebHLH18* actively regulates low temperature-induced leaf abscission in cassava. The cassava transgene showed that the overexpression strain slowed the low temperature-induced cassava leaf abscission rate. Further studies showed a connection between the decrease in the rate of cassava leaf abscission caused by the overexpression strain and the increase in POD activity. Moreover, Genome-wide association studies (GWAS) analysis showed that the single nucleotide polymorphism (SNP) variation in the promoter region upstream of *MebHLH18* led to the change of *MebHLH18* expression, increased POD activity, and decreased the rate of leaf abscission in cassava. Our results show that the natural variation of the promoter region of *MebHLH18* increases the antioxidant levels at low temperatures and decreases the low temperature-induced leaf abscission rate.

## Materials and methods

### Plant material

This study used 170 cassava germplasms in total. Please refer to **Table S3** for a detailed list of material names.

### Low-temperature stress experiment and physiological index determination

The cassava planting method was carried out according to Liao methods (Liao et al., 2016a). In brief, the stems of cassava wildtypes and transgenic plants were cut into with uniform length, planted in flowerpots mixed uniform medium with an equal amount of soil and sand, and cultured in the rain-proof shed. For low-temperature stress treatments, four-month-old cassava plants with a uniform growth status were chosen for low-temperature stress experiments, each pot contained 3 cassava plants and 4 pots were prepared for 1 treatment and repeated 3 times, 1 treatment with 3 times repetition regarded as one biological replicate, the selected cassava plants with relatively consistent growth potential were selected to be cultured at 4°C for low-temperature treatment, while the same number of cassava plants cultured at room temperature were identified as the control group. After the plants were treated at 4°C for 24 hours, the control plants were placed in the greenhouse for normal growth. The treated and control plants were placed in the greenhouse simultaneously for growth recovery. Samples collection and phenotypes detection were carried out after 10 days of growth recovery. The Wang method was used to detect the physiological indexes of SOD, POD, and CAT activities (Wang et al., 2022). In detail, the upper, middle, and lower leaves of the plants were selected, cut, mixed, frozen in liquid nitrogen, and stored at −80°C to detect the physiological index. The SOD activity was determined using the nitroblue tetrazolium reduction method and the NBT method. The POD activity was detected using the guaiacol method. The CAT activity was examined by the H_2_O_2_ ultraviolet absorption method following the corresponding reagent box operation instruction of Suzhou Keming Company (Wang et al., 2022).

### Gene subcellular localization

The method and process of cassava gene subcellular localization are described above. The gene expression vector was constructed and transformed into the GV3101 strain. Transformation and infection tests were carried out on tobacco leaves that had been grown for six weeks. Confocal microscopy (Olympus FV1000) was used to observe fluorescence signals after two days of conversion (Xiong et al., 2018).

### Phylogenetic analysis

The neighbor connection method and bootstrap analysis were used to analyze the phylogenetic tree constructed by MEGA6 (500 replicates). The online tool Evolview was used to visualize the phylogenetic tree (Wang et al., 2022). We used ClustalW for multiple sequence alignment.

### Real-time polymerase chain reaction

Real-time RT-PCR was used to verify the expression analysis of *MebHLH18.* The procedure of real-time PCR refers to the method published by Wang et al. (Wang et al., 2022). To analyze the expression levels of *MebHLH18* under low-temperature stress treatments, Leaf abscission zones were harvested at 0, 6, 12, 24, 48, and 72 h after 4°C treatments, the leaf abscission rate was also calculated at the same time points after low-temperature stress treatments, POD activities were also detected in both leaves and root at the selected time points after low-temperature stress treatments. For analysis the expression levels of *MebHLH18* in cassava genotypes SM2300-1 and COL514, the leaf abscission zones were harvested at 12 and 24h after 4°C treatments. The primers list in the **Table S6**. The RNA of three independent biological samples was reverse transcribed and used for real-time analysis.

### Population genetic analyses

GEMMA was adopted for GWAS analysis (http://www.xzlab.org/software.html). The software was used for analysis, and the mixed linear model was used to correct the population structure and the genetic relationships between the individuals. GEMMA was used to perform association analysis based on the correlation of different cassava populations, and potential candidate SNPs were screened based on the significance of the association (P-value). LDBlockShow software was used to draw the haplotype block.

### Measurement of leaf abscission rate induced by low temperatures

10 days of growth recovery after low-temperature treatment, the leaves of 170 representative cassava germplasms were carefully counted. The leaves of five cassava plants were statistically analyzed for each cassava germplasm. The correlation analysis was based on the final number of leaves growth and the average number of leaves of five plants.

### Plasmid construction and cassava transformation

The *MebHLH18* gene transformation vector was created using the same construction and gene transformation methods as Wang et al. (Wang et al., 2022). The expression cassette of cassava *MebHLH18* was inserted into the binary vector pCAMBIA1301 under the control of the CaMV 35S promoter to generate 35S::*MebHLH18* containing the hygromycin phosphotransferase gene (*hpt*). The construct was introduced into *A. tumefaciens* strain LBA4404 and then used for genetic transformation. The embryogenic callus of cassava TMS60444 and *A. tumefaciens*-mediated genetic transformation were performed as described by Xu et al. (2013a). To construct the RNAi plasmid, a cDNA fragment of *MebHLH18* was amplified from cassava and inserted into the vector pCAMBIA1301 as described previously (Wang et al., 2022). The primers for *MebHLH18* gene transformation vector construct were list in **Table S6**.

### Promoter sequence analysis

Fisher’s exact test was used to compare sequence variation and low temperature-induced leaf abscission (Jung, 2014).

### Transient expression assay of promoter activity

The cassava varieties SM2300-1 and COL514 were used to clone the 2-kb promoter fragment upstream of *MebHLH18*. The SNP mutation fragment from nucleotides T to A was performed by overlapping extension PCR (**Table S6**). All constructed fragments were inserted into the pNC-Green-LUC vector. Protoplasts were isolated from the leaves, and each *MebHLH18* promoter LUC gene fusion construct was used to transform the protoplasts transiently. LUC to REN luciferase activity was measured using a dual luciferase reporting analysis system (Promega) (Huo et al., 2017). The primers used are listed in **Table S1**.

### Histochemical staining and detection of ROS

H_2_O_2_ and O_2_·- levels were determined using the method published by Geng et al. (Geng and Liu, 2018). 3,3’-diaminobenzidine (DAB) and NBT were adapted respectively to analyze H_2_O_2_ and O_2_·- levels, Briefly, the cassava leaves were placed in freshly prepared solutions of DAB (1 mg ml^–1^ in 50 mM potassium phosphate, pH 3.8) or NBT (1 mg ml^–1^ in 50 mM potassium phosphate, pH 7.8). After incubation for 12 h in the dark at room temperature, the chlorophyll was removed with 75% ethanol in a boiling water bath and the leaves were then photographed (Geng and Liu, 2018).

### Statistical analysis

Low-temperature treatment was repeated three times for each line. All data were statistically evaluated using Statistical Package for Social Sciences software (SPSS statistics). Statistical difference was determined by analysis of variance based on Fisher’s LSD test. P < 0.05 was considered statistically significant.

## Results

### The levels of reactive oxygen scavengers vary significantly in different cassava genotypes with low temperature-induced leaf abscission

**Figure 1:**
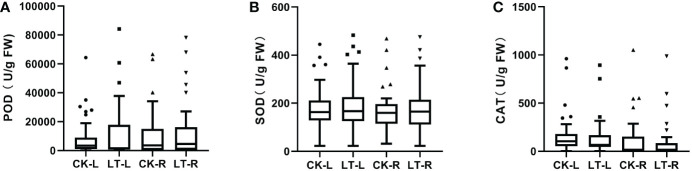
The levels of reactive oxygen scavengers significantly different among cassava genotypes during low temperature-induced leaf abscission. **(A–C)** Peroxidase (POD) **(A)**, Superoxide dismutase (SOD) **(B)** and Catalase (CAT) **(C)** activities in cassava were detected among cassava genotypes during low temperature-induced leaf abscission. CK-L: Leaf control; LT-L: Leaf treated at 4°C for 24 hours; CK-R: root control; LT-R: root treated at 4°C for 24 hours. In low temperature-induced cassava leaf abscission, significant differences in reactive oxygen species scavengers were detected between cassava genotypes. Cassava grown for 120 days was selected as the experimental material. The cassava plants with consistent growth were selected for low-temperature treatment. After the plants were treated at 4°C for 24 hours, the control plants were placed in the greenhouse for normal growth. The treated and control plants were placed in the greenhouse simultaneously for growth recovery. Samples were taken after 10 days of growth recovery. The leaves were collected from the upper, middle, and lower parts of the four plants. The roots of the four plants were mixed and frozen in liquid nitrogen and stored at −80°C. During low temperature-induced leaf abscission, POD, SOD and CAT activities in cassava were detected. The value is represented as mean ± standard error (n=40).

Our previous studies confirmed that ROS and ROS scavengers significantly regulate stress-induced leaf abscission in cassava (Liao et al., 2016a; Liao et al., 2016b). To study the reactive oxygen scavenger levels in different cassava genotypes under low temperature-induced leaf abscission, we measured the levels of reactive oxygen scavengers (SOD, CAT, and POD) in the roots and leaves of different cassava genotypes under low temperatures (**Figure 1**; **Table S1**). The results showed significant differences in the natural changes of reactive oxygen scavengers in different cassava genotypes during low temperature-induced leaf abscission. POD content in the leaves was the highest (84236 U/g FW), while that in the roots was the lowest (83.77 U/g FW), CAT content in the roots was the highest (1050 U/g FW), while that in the roots and leaves was the lowest (1.9 U/g FW), SOD content in leaves was the highest (482.7 U/g FW), while SOD content in leaves was the lowest (22 U/g FW) (**Figure 1**; **Table S1**). Among the three antioxidants induced by low temperature, the variation of POD was the most obvious. As shown in **Figure 1** and **Table S1**, the reactive oxygen scavengers of different cassava genotypes exhibit continuous quantitative characteristics and obvious changes during low temperature-induced leaf abscission. The maximum value of a reactive oxygen scavenger is POD-L, and the minimum value is CAT-L and CAT-R (**Figure 1**; **Table S1**). The results showed that low temperature-induced leaf abscission caused the ROS scavenger content in the leaves of different cassava genotypes to differ significantly.

We carried out descriptive statistical analysis on the phenotypic data of characters obtained from low-temperature stress experiments to study the effects of low-temperature stress on cassava ROS scavengers. As shown in **Table S1**, Low-temperature stress increases the diversity of cassava population ROS scavengers, the variation coefficients of POD content measured in the leaves and roots were higher in the treatment group than in the control group. CAT had the highest coefficient of variation of the measured values of the traits under the control and cold treatment conditions (the average coefficient of variation is 213.8). SOD had the lowest coefficient of variation (the average coefficient of variation is 50.46).

This study conducted variance analysis on the data of the low-temperature group and control group in the same part of the same experiment to further understand whether low-temperature stress significantly impacts cassava the ROS scavenger. The results are shown in **Table S1**. The value of POD in the leaves group was significantly higher than that in the root group (P=1.0E-4, leaf, P=0.6476, root). The average value of the POD population in the treatment group was significantly higher than that of the control group (P<0.001). These results showed that different cassava materials had different responses to low-temperature stress and that there were obvious common characteristics among cassava materials, this shows that the characteristics of active oxygen scavengers vary greatly in cassava germplasm under low temperature stress, especially the variation of POD is very rich.

### *MebHLH18* expression in the root is highly related to POD expression in the root and leaf in low temperature-induced leaf abscission process in cassava

**Figure 2:**
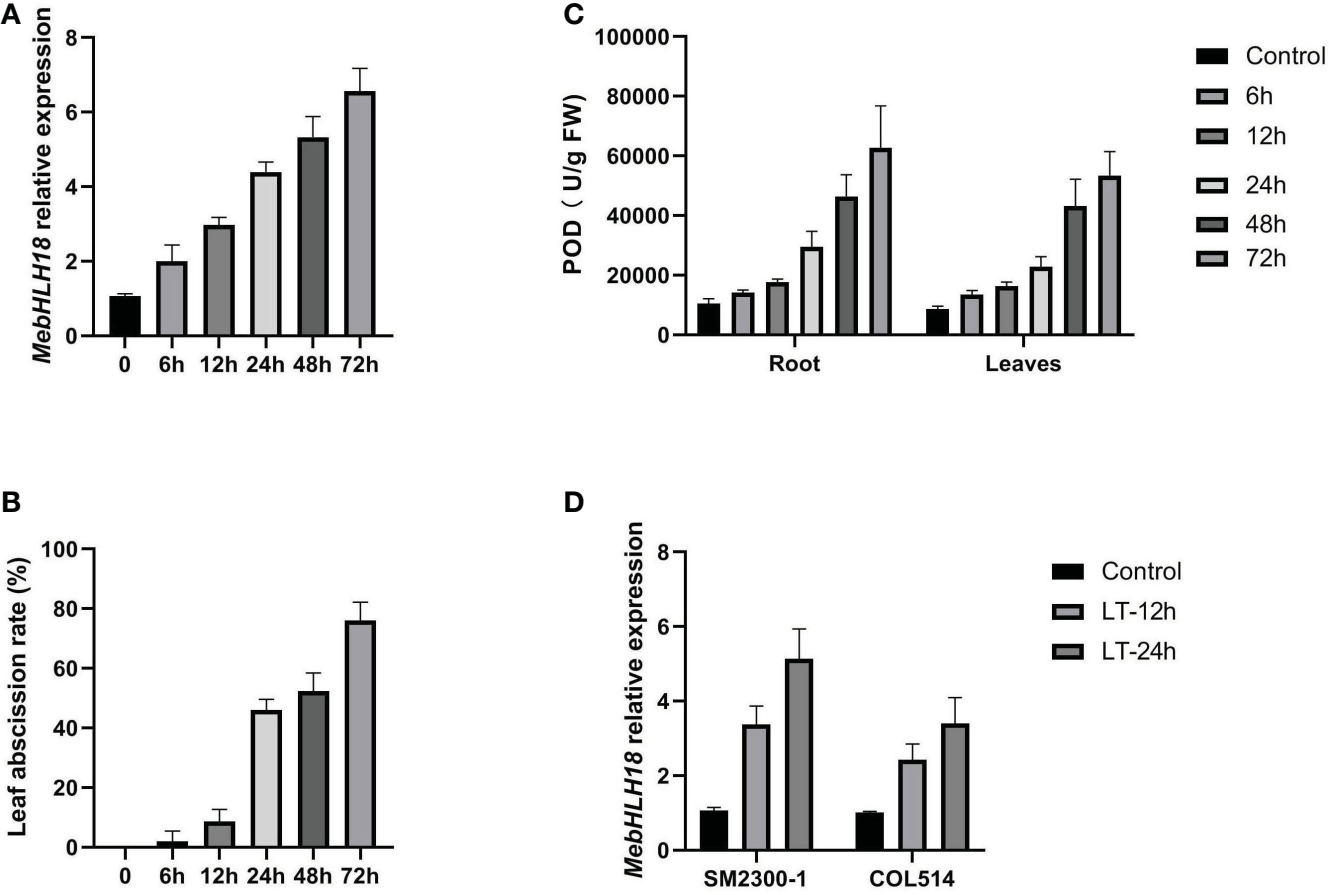
The expressions of *MebHLH18*, POD levels as well as leaf abscission displayed similar pattern under cold treatment. **(A)** The *MebHLH18* expression levels in low temperature-treated (4°C) (0h, 6h, 12h, 24h, 48h and 72h) plants were analyzed by fluorescence real-time quantitative polymerase chain reaction. **(B)** Leaf abscission rates were analyzed at 10 days recovery growth after low temperature-treated for six time points selected in **(A)**. **(C)** POD level analysis in low temperature-treated (4°C) (0h, 6h, 12h, 24h, 48h and 72h) plants were analyzed. **(D)** The *MebHLH18* expression levels in SM2300-1 and COL514 cassava genotypes under low temperature treated for 12h and 24h. Cassava grown for 120 days was selected as the experimental material. The cassava plants with consistent growth were selected for low-temperature treatment. After the plants were treated at 4°C for 24 hours, the control plants were placed in the greenhouse for normal growth. The treated and control plants were placed in the greenhouse simultaneously for growth recovery. Samples were taken after 10 days of growth recovery. Five plants selected as one samples, each sample was replicated four times. The value is represented as mean ± standard deviation (n=20) in **(A–D)**.

Our previous studies confirmed that stress-induced genes significantly regulate cassava leaf abscission (Liao et al., 2016a; Liao et al., 2016b). We analyzed the correlation between *MebHLH18* expression and low temperature-induced leaf abscission to study the regulatory genes involved in low-temperature-induced leaf abscission. Quantitative PCR was used to examine the expression pattern of *MebHLH18* in the root at room temperature and 4°C for six time points (0h, 6h, 12h, 24h, 48h and 72h). The results showed that *MebHLH18* was induced expression by low temperature treatment in six time-points (**Figure 2A**). Moreover, the expression levels of *MebHLH18* were higher and higher with the prolongation of low temperature treatment (**Figure 2A**). The leaf abscission rates were analyzed at 10 days recovery growth after low temperature-treated for the six time points, the results indicated that the leaf abscission rates highly related to the *MebHLH18* expression covering the six time points by correlation analyses (**Figure 2B**; **Table S2**, Correl=0.97). We also analyzed the correlation between *MebHLH18* expression and POD activity. The results are as shown in the **Figure 2C**, *MebHLH18* expression in roots is significantly positively correlated with POD activity (**Figure 2B**; **Table S2**, Correl=1.00). Roots exhibit high levels of *MebHLH18* expression. The roots and leaves exhibit the highest levels of the corresponding POD activity, indicating that *MebHLH18* may be related to the POD activity induced by low-temperature stress.

To understand the *MebHLH18* patterns in different cassava genotypes, the expression patterns of *MebHLH18* in SM2300-1 and COL514 were carried out. The results showed that *MebHLH18* was induced to express in both germplasms at low temperatures, with higher expression in SM2300-1 and lower expression in COL514 (**Figure 2D**). These results indicate that the expression of *MebHLH18* is different in different cassava germplasms under low-temperature stress.

### *MebHLH18* is a *bHLH* family member

**Figure 3:**
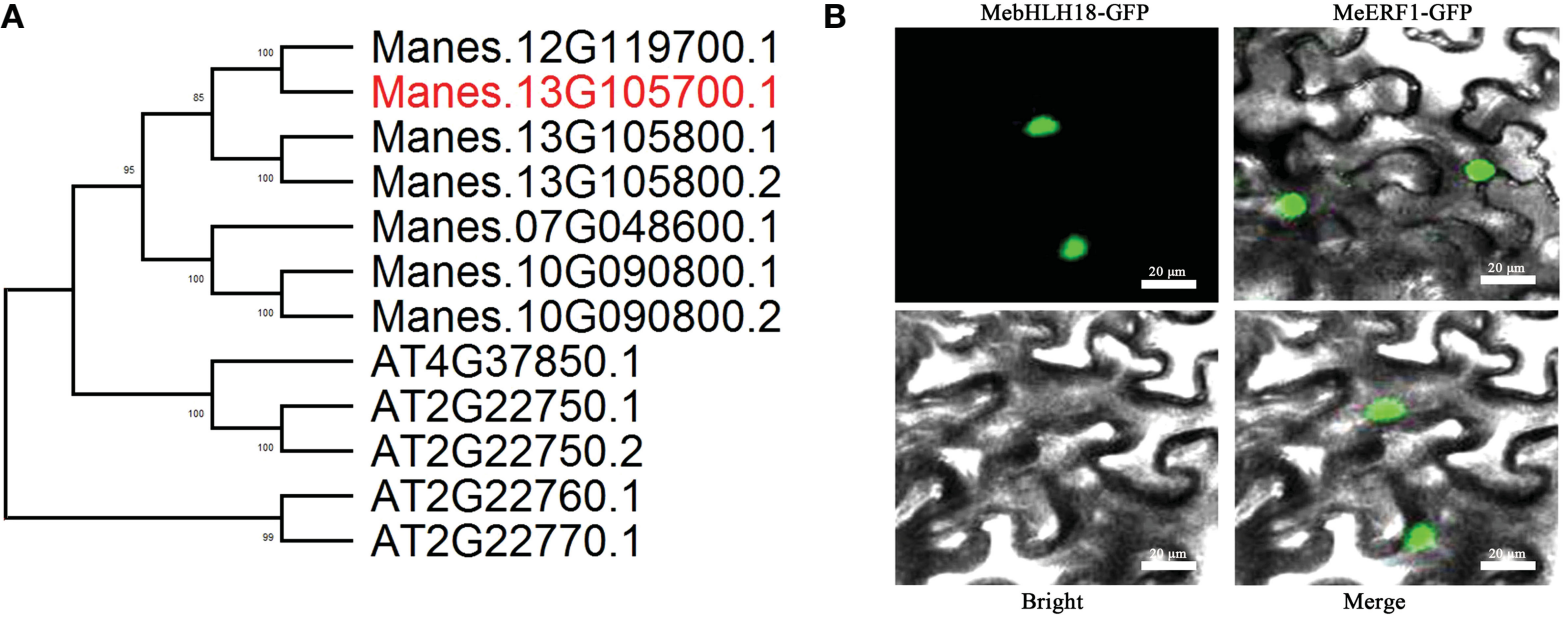
Functional characterization of *MebHLH18.* **(A)** Phylogenetic comparison of group indicated in **Figure S1** (marked in red) bHLH members in cassava and *Arabidopsis*. Bootstrap values from 500 resamplings are indicated. **(B)** Subcellular localization of *MebHLH18*. Transient expression of 35S:*MebHLH18-GFP* and 35S:*MeERF1-GFP* in *Nicotiana benthamiana* leaves is shown. *MeERF1-GFP* was used as a nuclear marker. Bars, 20 μm.

All the *bHLH* gene sequences of cassava genotype AM560-2 were obtained from the JGI cassava genome database. Phylogenetic comparison of *bHLH* members in cassava and *Arabidopsis* was carried out (**Figure S1**; **Figure 3A**). The *MebHLH18* (Manes.13G1057100) gene region is 1400 bp long, including two introns (89 bp and 101 bp, respectively) and three coding exons (677 bp, 374 bp, and 74 bp, respectively). The CDS sequence of the *MebHLH18* gene is 1125 bp long, encoding 374 amino acids. The CDS sequence is aligned in NCBI conservative domain database. A *bHLH* domain was found at the N-terminal of *MebHLH18*, indicating that the *MebHLH18* gene may be a member of the *bHLH* family. *MebHLH18* was confirmed to be a nuclear localization protein in tobacco leaves by infiltrating *Agrobacterium tumefaciens* (strain GV3101) containing 35S: *MebHLH18* GFP with 35S: *MeERF1* GFP as a positive control (**Figure 3B**).

### Overexpression of *MebHLH18* in cassava decreases the low temperature-induced leaf abscission rate by increasing POD levels

**Figure 4:**
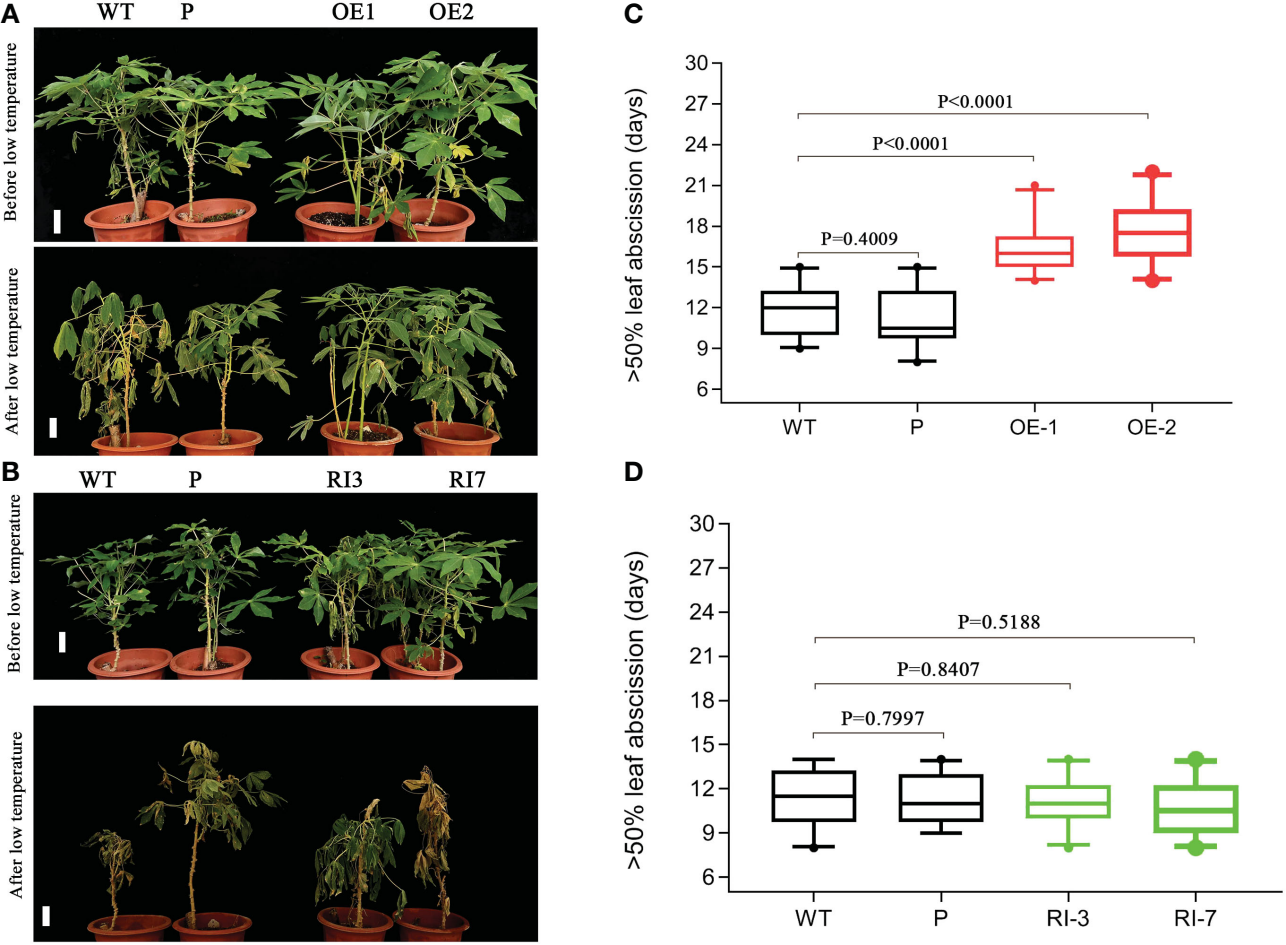
*MebHLH18* transgenic cassava plants showed that *MebHLH18* expression alleviated leaf abscission exposed to low temperatures. **(A)** *MebHLH18* overexpression lines (OE), wild-type plants (WT) and overexpression empty vector lines (P) recovered 10 days after a 4°C low-temperature treatment exposure. **(B)** *MebHLH18* RNAi lines (RI), wild-type plants (WT) and overexpression empty vector lines (P) recovered 10 days after a 4°C low-temperature treatment exposure. **(C)** The leaf abscission in overexpressing *MebHLH18* lines, WT, and overexpressing empty vector showing the time (day) until >50% of leaves had abscised after a 4°C low-temperature treatment exposure. **(D)** The leaf abscission in knockdown, WT, and overexpressing empty vector showing the time (day) until >50% of leaves had abscised after a 4°C low-temperature treatment exposure. All samples were collected from 120-day-old cassava plants for analysis. The data in the graph is expressed as mean ± standard error based on four technical replicates (n=20). Bar=50 mm. Mann Whitney test based on ranks in **(C, D)**.

We obtained *MebHLH18* transgenic plants with overexpression and RNA interference through cassava transgenic to know whether the biological function of *MebHLH18* is related to low temperature-induced leaf abscission. Transgenic cassava lines were identified by hygromycin screening and real-time RT-PCR (**Figure S2**). *MebHLH18* was expressed at significantly higher levels in overexpression lines (OE1, OE2, and OE6) compared to wild-type and *MebHLH18* RNA interference lines (RI3, RI5, RI7) (**Figure S2**). Two transgenic lines (OE1 and OE2) and two RNA interference transgenic lines (RI3 and RI7) were selected for further analysis under the condition of low temperature-induced leaf abscission. However, there is no significant difference between the growth of transgenic plants and wild-type plants at room temperature (**Figures 4A, B**). However, the difference is significant when low-temperature stress causes leaf abscission (**Figures 4A, B**). We analyzed the days of more than 50 percent the leaf abscission in *MebHLH18* overexpressing lines, *MebHLH18* RNA interference lines, and wild type at 10 days recovery growth after low-temperature treatment. The results showed that when these plants were exposed to low-temperature stress, the overexpressing plants delayed leaf abscission for about 5-6 days as compared to the wild type plants (**Figure 4C**), while in transgenic plants silenced for *MebHLH18* expression leaf abscission rate have no different from wild type plants (**Figure 4D**), resulting in decreased leaf abscission in *MebHLH18* overexpressing plants lines as compared to knockdown plants and wild type (**Figures 4C, D**). The extent of leaf abscission negatively correlated with *MebHLH18* expression in knockdown and over-expressing lines (**Figures 4C, D**). These results indicate that *MebHLH18* regulates the abscission of cassava leaves induced by low-temperature stress.

**Figure 5:**
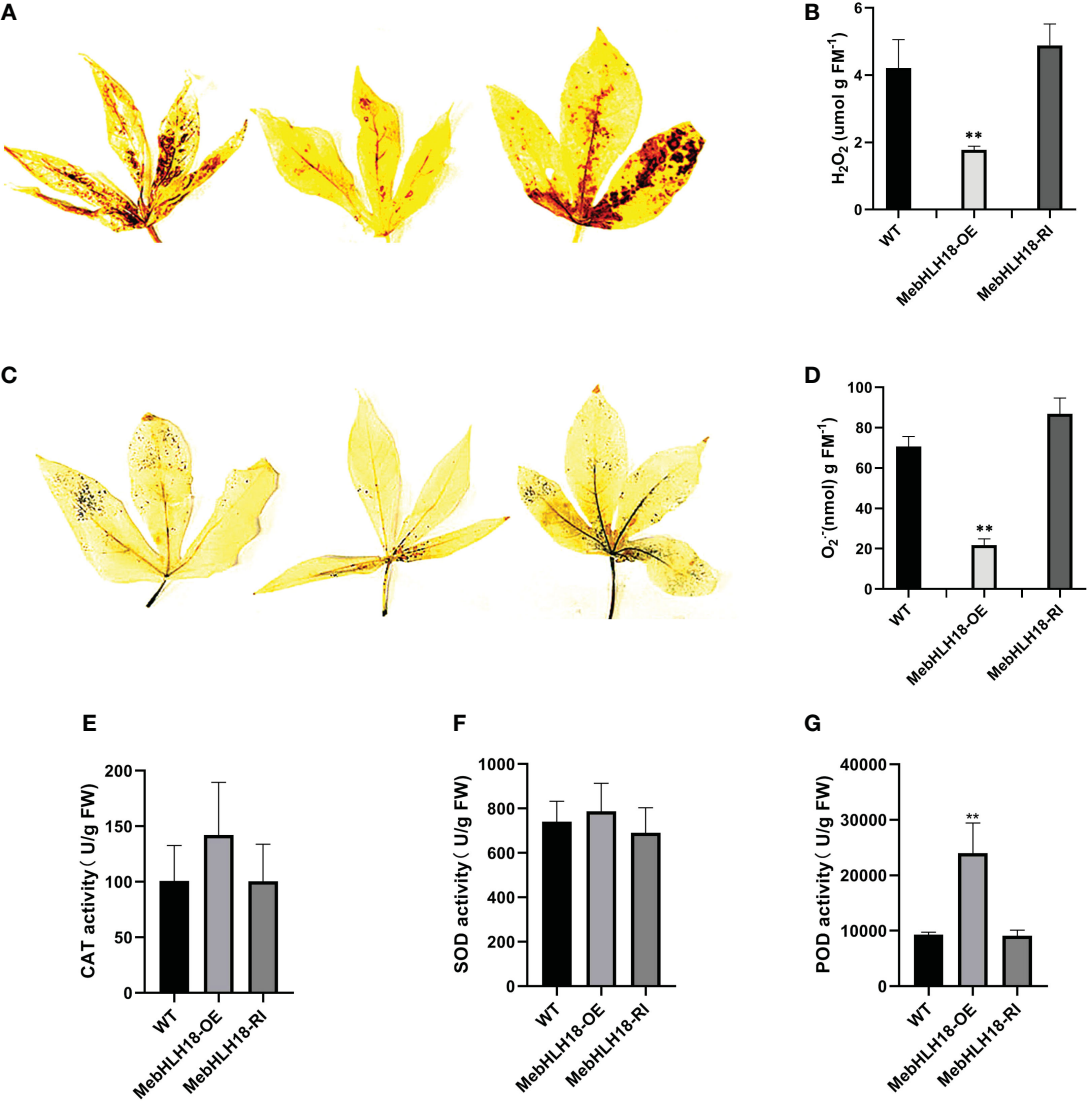
Reactive oxygen species and antioxidant enzyme activities levels in wild-type plants and transgenic lines. **(A)** Histochemical staining of diaminobenzidine was used to detect the *in situ* accumulation of H_2_O_2_ in the transgenic lines and WTs after low-temperature treatment. **(B)** Histochemical staining of nitro blue tetrazolium was used to detect the *in situ* accumulation of O_2_·- in the transgenic lines and WTs after low-temperature treatment. **(C)** Quantitative H_2_O_2_ measurements in transgenic lines and WT plants. **(D)** Quantitative O_2_·- measurements in transgenic lines and WT plants.The error bar represents three independently copied standard deviations. The asterisk indicates a significant difference between WT and transgenic lines (**P < 0.001). **(E–G)** Activity of catalase (CAT) **(E)**, superoxide dimutase (SOD) **(F)**, and peroxidase (POD) **(G)** in transgenic lines and WT after low-temperature treatment. The asterisk indicates a significant difference between WT and transgenic lines (**P < 0.001).

Our previous studies have shown that stress-induced ROS plays a key role in regulating cassava leaf abscission (Liao et al., 2016a). We analyzed the ROS level in transgenic lines and wild types because the leaf abscission rate of transgenic cassava lines overexpressing *MebHLH18* is significantly lower than that of wild type and interference lines. The diaminobenzidine (DAB) and nitro blue tetrazolium (NBT) histochemical staining results showed that compared with the overexpression transgenic plants, the leaves of wild plants and interference lines were darker after DAB and NBT staining (**Figures 5A, B**), indicating that the cell levels of H_2_O_2_ and O_2_·- of transgenic plants were lower than those of non-transgenic plants (**Figures 5A, B**). The histochemical staining and determination results showed that the transgenic overexpression lines accumulated low levels of ROS under low-temperature stress (**Figures 5C, D**).

We analyzed the reactive oxygen scavengers (SOD, POD, and CAT) in overexpression lines, interference lines (**Figures 5E–G**), and wild-type plants to understand whether reactive oxygen scavenger of the plant plays a role in regulating *MebHLH18* in the abscission of cassava leaves induced by low-temperature stress. We analyzed the activity of ROS scavengers in transgenic and non-transgenic lines. Under low-temperature conditions, POD activity in overexpression lines was significantly higher than in wild-type and interfering lines (**Figure 5G**). The difference in POD activity confirmed POD that plays important role in regulating low temperature-induced leaf abscission.

### Natural variation in the promoter of *MebHLH18* is associated with leaf abscission in cassava under low temperature

**Figure 6:**
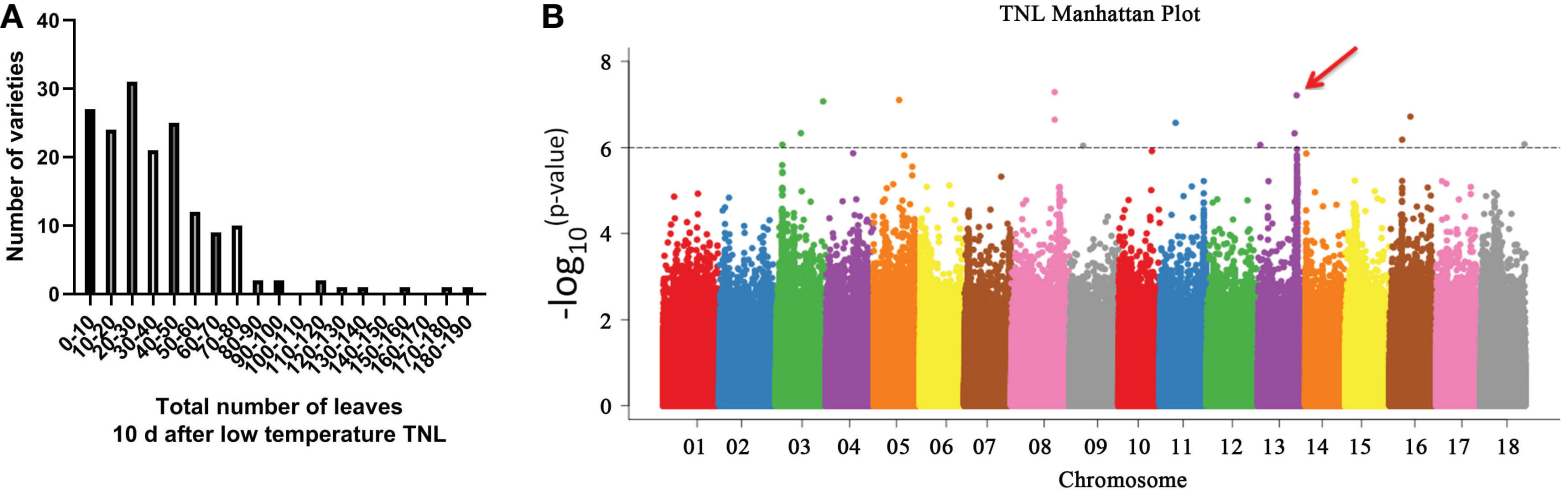
Genome-wide association studies (GWAS) analysis revealed that *MebHLH18* is involved in low temperature-induced leaf abscission. **(A)** Frequency distribution of the total number of leaves at 10 days after low temperature (TNL). **(B)** Manhattan map of the TNL phenotype. Six quantitative trait loci (QTLs) had TNL values greater than the threshold of significance induced by low temperature-induced leaf abscission. The GWAS peak on chromosome 13 is located in a QTL, Manes. 13G105700 (*MebHLH18*), which has a peak value. The red arrow indicates the location of the *MebHLH18* gene.

We used 170 cassava germplasms (**Table S3**) from China, Colombia, and Brazil to determine the natural variation of genes related to low-temperature control. We measured five low-temperature traits (**Table S4**) using these germplasms as research objects, and we used the number of growing leaves (TNL) measured 10 days after low temperature as a representative trait (**Figure 6A**; **Figure S3**). The low temperature-induced leaf abscission phenotypes of these germplasms were analyzed. The analysis of the number of leaves with various qualities growing 10 days after low temperature revealed that the minimum number of leaves growing after the low temperature was 0, and the maximum number of leaves was 190. The number of leaves growing for most germplasms was 0-80, indicating that the number of leaves with various qualities growing under low temperature showed great variation (**Figure 6A**). Among them, SM2300-1 retained many leaves after low-temperature treatment, while COL514 almost lost all its leaves. We sequenced the whole genome of these 170 cassava varieties to detect nucleotide polymorphism. Then, we performed a GWAS analysis of TNL in these cassava populations using a mixed linear model and kinship correction (Huang et al., 2010; Yano et al., 2016). As shown in **Figure 6**, the TNL value of GWAS as a candidate for low-temperature response shows that the quantitative trait loci on six chromosomes (chromosomes 3, 5, 8, 11, 13, and 16) exceed the significance threshold (**Figure 6B**; **Table S5**). We analyzed these GWAS peak SNP sites and found that the GWAS peak was the highest in chromosome 13 (**Figure 6B**; **Table S5**). Further, we analyzed the GWAS peak site in this chromosome and found that this site belongs to the promoter region of *Manes.13G105700* (*MebHLH18*) gene.

**Figure 7:**
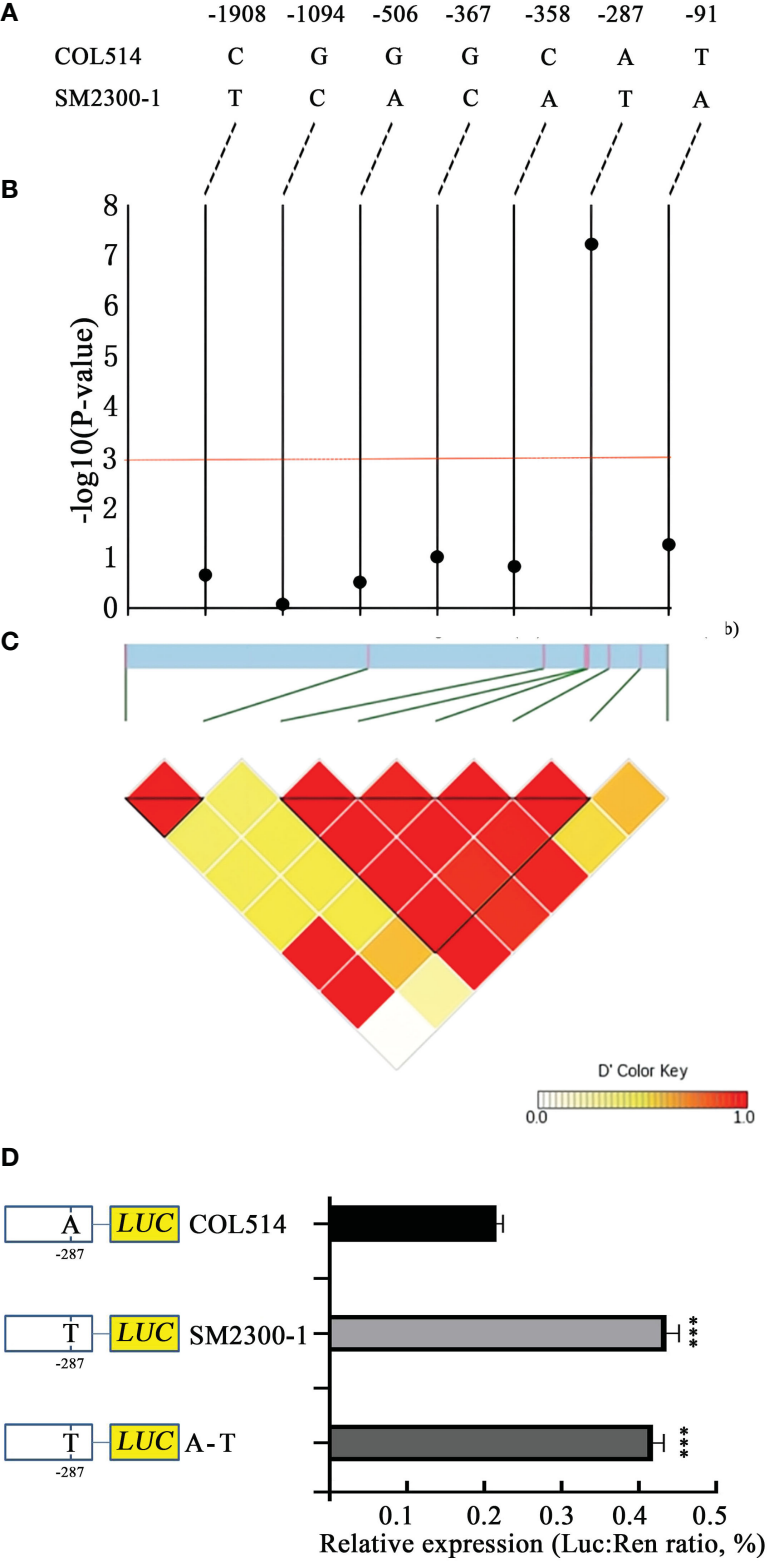
Association test and expression level of the *MebHLH18* variant. **(A)** Sequence comparison of the bHLH18 promoter region in cassava germplasm COL514 and SM2300-1. **(B)** A correlation test of seven variants in the 2.0 kb promoter region with leaf abscission. The black dots represent seven variations. **(C)** A triangular matrix with a pair of linkage disequilibrium. **(D)** Transient expression analysis of three single nucleotide polymorphism effects in the *MebHLH18* promoter (n=5). The value is represented as mean ± standard deviation. Student t-test was used to generate P-value; ***P < 0.001.

The promoter region of the *13G105700 (MebHLH18)* gene was analyzed. Although the coding region of the *MebHLH18* genomic DNA did not differ between the parents SM2300-1 and COL514, there were seven different DNA site variations in the 2.0 kb region upstream of the starting site (**Figure 7A**). We inferred this region from 170 cassava varieties to determine the relationship between low temperature-induced leaf abscission and the *MebHLH18* promoter region (**Table S5**). A strong signal was detected at the -287 site of the promoter region (GWAS peak value was 7.21, **Table S5**; **Figure 7B, C**). It was found that after exposure to low temperature, there were more leaves and T in the promoters of most varieties. In contrast, fewer leaves and A are the promoters of most varieties after exposure to low temperature. Therefore, the A-T transformation of the promoters may cause differences in gene expression, which cause changes in the leaf growth number of cassava varieties after exposure to low temperature. We inserted the mutant promoter fragment into the binary vector pNC-Green-LUC and transiently expressed it in the protoplast to determine whether this SNP affects the expression of *MebHLH18*. The 287 SNP mutation (1 construction, A is T) in the mutant COL514 promoter significantly enhanced its relative expression when compared to the COL514 promoter (**Figure 7D**). To further confirm its role in regulating *MebHLH18* expression, we compared the expression levels of genes with SNP sites for T and A. The 287 T genotype expresses *MebHLH18* at a relatively higher level than 287 A (**Figure 7D**). These results indicate that changing its transcriptional expression level in cassava requires the conversion of 287 SNP upstream of it.

**Figure 8:**
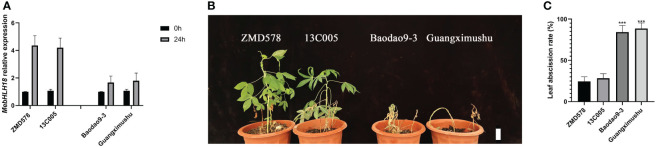
The A-T conversion of the *MebHLH18* promoter region caused the change in *MebHLH18* expression in different cassava germplasms. **(A)** The expression analysis of germplasms ZMD578 and 13C005 (with the same single nucleotide polymorphism (SNP) as SM2300-1), Baodao9-3, and Guangximushu (with the same SNP as COL514). The selected cassava germplasms were treated at 4°C for 24 hours under the same conditions. Values are represented as mean ± standard error (SE) (n=4). **(B, C)** Different SNP cassava germplasms, ZMD578, 13C005, Baodao9-3, and Guangximushu, had different rates of leaf abscission. All materials were treated at 4°C for 24 hours and recovered for 10 days, and then leaf abscission analysis was conducted in **(C)**, with mean ± standard error (n=4). The Student’s t-test determined statistical significance. The asterisk indicates the statistically significant difference calculated using Student’s t-test: ***P < 0.001.

To further study whether the change of this site contributes to the tolerance of cassava to low temperatures, we used cassava as a material to study the activity of the *MebHLH18* promoter under T and A conditions. We compared the gene expression of cassava lines with the T promoter and cassava lines with the A promoter to determine whether this SNP affects the expression of *MebHLH18*. As expected, ZMD578 and 13C005 with T promoters are positively correlated with bHLH18 expression levels, while negatively correlated with Baodao9-3 and Guangximushu with A promoters (**Figure 8A**). Additionally, to determine whether this SNP affects low temperature-induced leaf abscission phenotype in cassava, we compared and studied the degree of leaf abscission of cassava germplasms with different loci after low-temperature treatment and analyzed the results. The results showed that cassava lines with different loci recovered after low-temperature treatment, and it was found that the leaf abscission rate with the promoter at locus A was 74.6%-96.2%. In contrast, the leaf abscission rate of cassava lines with the promoter at locus T was 18.7%-35.8% (**Figures 8B, C**), indicating that the variation of promoter locus of the *MebHLH18* gene caused the change of leaf abscission under low temperature stress in cassava.

## Discussion

There was no obvious regularity in the average value of the SOD and CAT enzyme activity scavenged by active oxygen. However, POD activity differed significantly among the different low-temperature resistant genotypes. The difference between the overall average values of SOD and CAT is insignificant due to the dual effects of most low-temperature resistance traits. They have a positive effect on low-temperature resistance under mild stress but a negative effect or even the opposite trend under moderate low-temperature stress. This is mainly due to the differences in genotypes and antioxidants (Obidiegwu et al., 2015).

The reactive oxygen signal is the main regulating signal in cassava leaf abscission. However, there are several active oxygens, including O_2_·-, H_2_O_2_, and OH-, but it is unclear which one regulates the leaf abscission of cassava. Our research believes that reactive oxygen has a dual role in regulating cassava at low temperatures; specifically, the early stages of low temperature can cause the defense response in cassava and increase its adaptability to low temperatures. However, the level of active oxygen will rise steadily as the low-temperature stress progresses. In contrast, the high level of active oxygen will attack the cell membrane and destroy the protein, lipid, and other cell components so that the defense barrier will be damaged. The cassava cells will be damaged, causing the leaves to fall off prematurely. Antioxidant determination results supported this finding. At low temperatures, antioxidant POD levels increased steadily, especially during the late stage of low-temperature stress. Significant increases in POD levels show that POD was the main antioxidant of cassava against low-temperature stress. POD is related to the elimination of O_2_·-, H_2_O_2_, and · OH- (Gechev et al., 2006), and our results are consistent with this conclusion. The antioxidant determination results showed that POD levels increased more significantly than other antioxidants during the low-temperature process, indicating that the removal of ROS caused by POD may be the main reason causing the different leaf abscission rates of cassava under low temperature stress. The literature has previously described the changes in POD regulated by bHLH18 under low temperatures, and our study confirmed this result. Moreover, the high levels of POD expression regulated by bHLH18 confer a degree of resistance to low-temperature stress. Our results further confirm that bHLH18 positively regulates POD under low temperatures. Cassava has a very obvious occipital abscission zone structure, which enables cassava to have a flexible mechanism of leaf abscission and growth transformation when exposed to severe stress conditions. ROS is the main regulatory signal of this mechanism. Our results show that when cassava is exposed to severe low-temperature stress conditions, the change in POD level caused by bHLH18 is a stress escape behavior. Under severe low-temperature stress, cassava regulates the shedding of some leaves in advance through ROS, returns some nutrients, and stores them in the cassava root tubers. Cassava can use the stored nutrients to grow new leaves after the low-temperature stress is relieved, which may be how it responds to stress or escapes it.

Many genome-wide association analysis methods are used to study plant trait changes. Changes in gene expression levels brought on by variations in the sequence of the promoter region will also change the traits of plants (Pan et al., 2015; Sun et al., 2020; Zhang et al., 2021). This is in addition to variations in the gene coding region, which affect how plants react to low temperatures. For example, Ruan et al. reported that the natural variation of the amino acids A and G in the TGW2 promoter determines the width and weight of rice grains (Ruan et al., 2020). Our research shows that bHLH18 expression increases when there is a 287 bp A to T substitution upstream of it. This increased bHLH18 expression results in a high level of POD expression, which decreases ROS and changes the abscission rate of cassava leaves at low temperatures. Our research shows that natural variation in the cassava promoter region is one of the reasons for the natural domestication and evolution of cassava.

## Conclusion

Here, we report that the transcription factor *MebHLH18* involved in regulating low temperature-induced leaf abscission in cassava. We found through GWAS analysis that the change in *MebHLH18* expression was caused by the SNP variation in the upstream promoter region of the gene. The overexpression of *MebHLH18* in cassava transgenic plants increased POD activity. It decreased leaf abscission rate, indicating that the single nucleotide variation in the *MebHLH18* promoter region led to the change in *MebHLH18* expression. The active oxygen scavenging system induced the decreased leaf abscission rate by low temperature.

## Data availability statement

The original contributions presented in the study are included in the article/**Supplementary Material**. Further inquiries can be directed to the corresponding authors.

## Author contributions

WL, SC and MP designed the experiments. WL, YW, YC, HX, JC, and MR performed the experiments. SC and MP analyzed the data. WL, SC and MP wrote the manuscript. All authors contributed to the article and approved the submitted version.
