# Supplementary material for: The transcription factor MebHLH18 in cassava functions in decreasing low temperature-induced leaf abscission to promote low-temperature tolerance
Source: Front Plant Sci. 2023 Feb 13;13:1101821. doi: 10.3389/fpls.2022.1101821 (PMC9968922; doi:10.3389/fpls.2022.1101821)
Supplement: Supplementary file 1 [file DataSheet_1.docx]

**The transcription factor *MebHLH18* in cassava functions in decreasing low temperature-induced leaf abscission to promote low-temperature tolerance**

**Wenbin Liao^1,2^**^*^**, Jie Cai^3^, Haixia, Xu^1,2^, Yilin Wang^1,2^, Yingjie Cao^1,2^, Mengbin Ruan^1,2^, Songbi Chen^3^**^*^ **and Ming Peng^1,2^**^*^

**SUPPORTING INFORMATION**


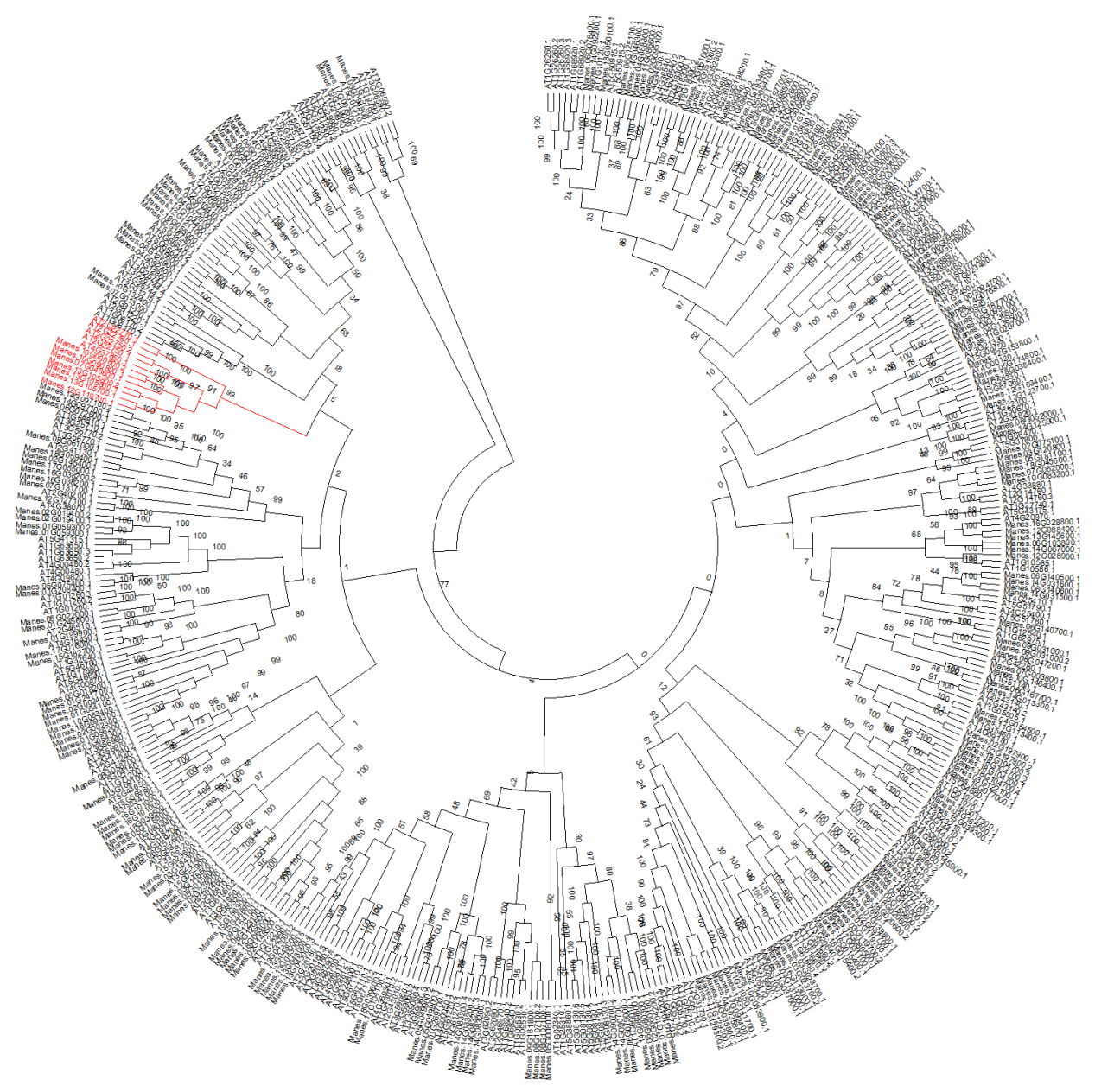


**Supplementary Figure S1 | Phylogenetic analysis of the *bHLH* transcription factors of *Arabidopsis* and cassava.**

Eighbor-joining phylogeny of *bHLH* genes of 2 species, as determined by MEGA5.0. Numbers on branches are bootstrap proportions from 500 replicates. The Red mark shows the group of *MebHLH18*.


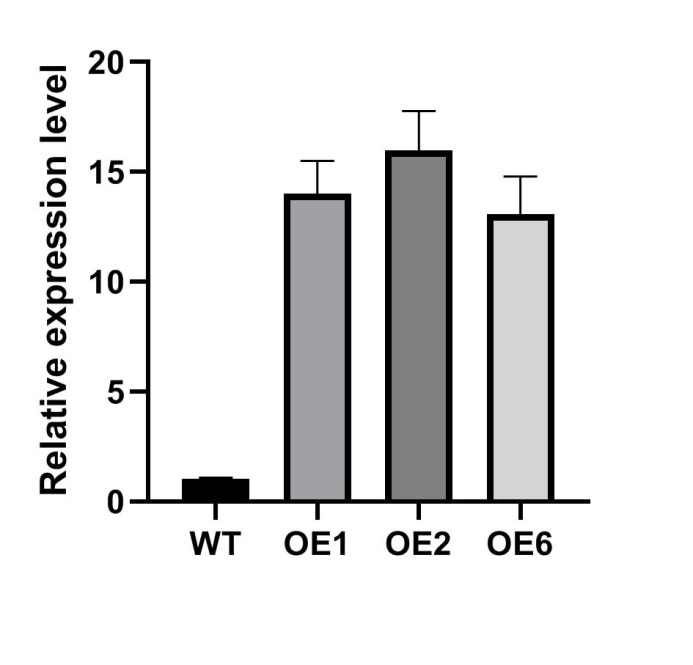


**A**

**B**


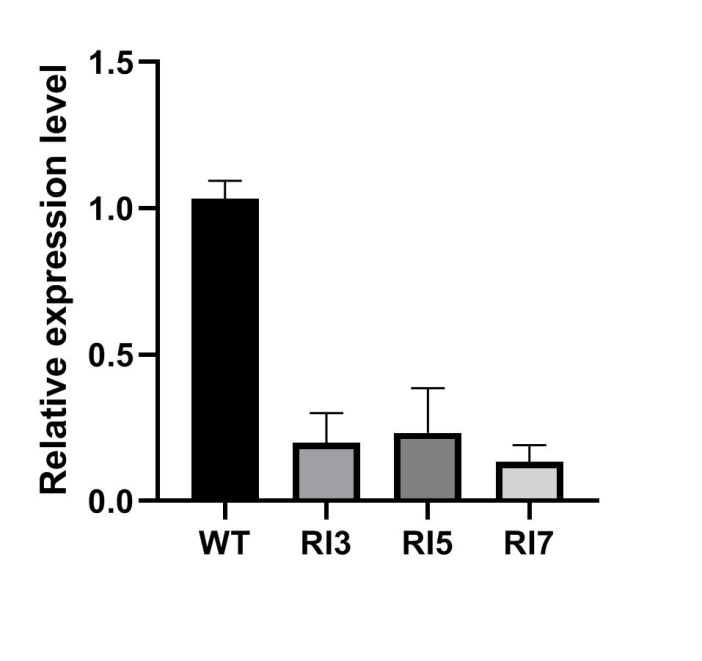


**Supplementary Figure S2 | Transgenic cassava lines were identified by real-time RT-PCR.**

The expression levels of *MebHLH18* in wildtype, overexpression lines (OE1, OE2, OE6) (**A**) and *MeBHLH18*-RNA interference lines (RI3, RI5, RI7) (**B**) were analyzed by RT-qPCR. Error bars indicate the SE based on three technical replicates. Two transgenic lines OE1 and OE2, and two RNA interference transgenic lines RI3 and RI7 were selected for futher analysis.

A


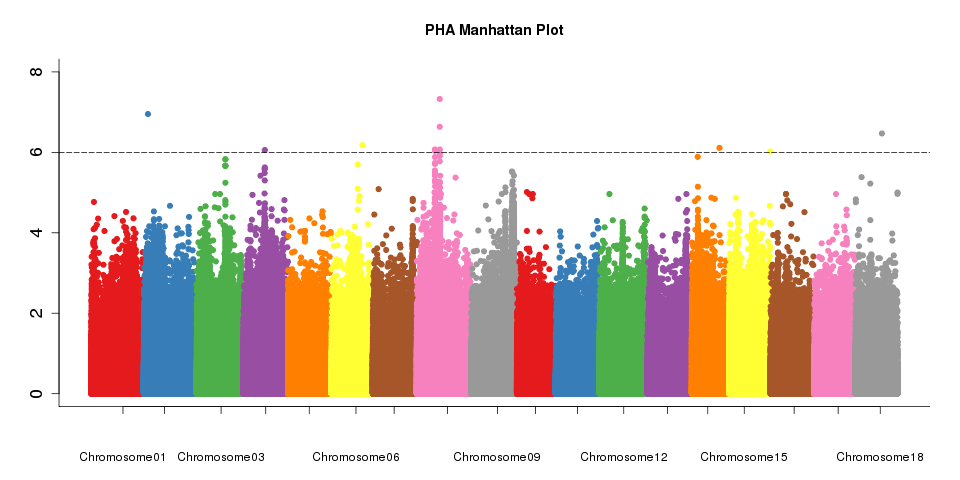


B


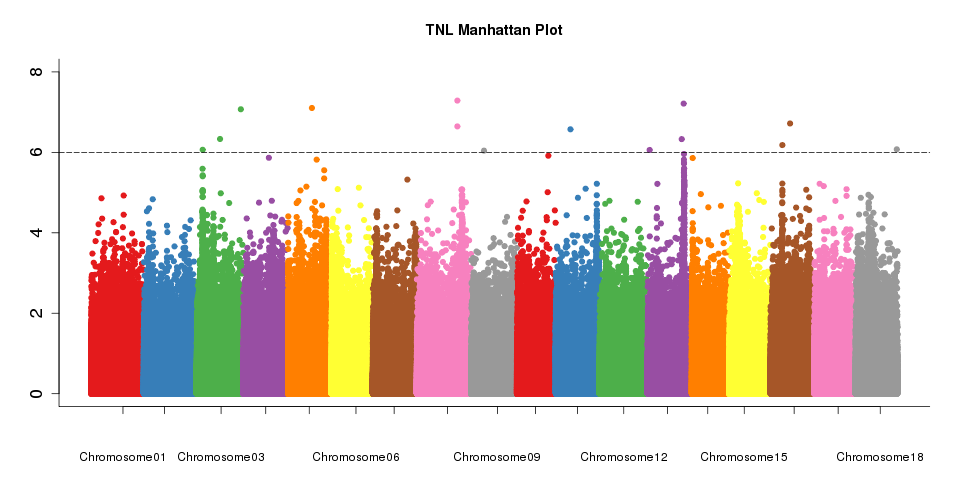


C


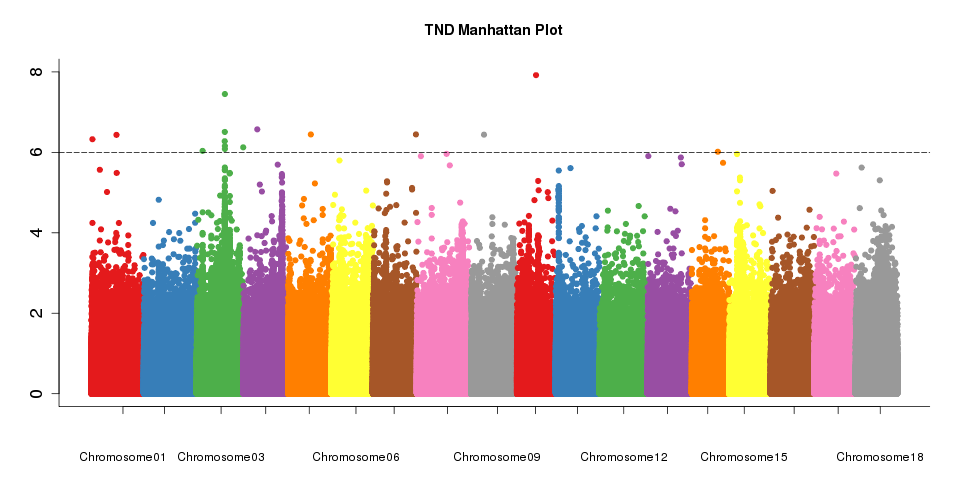


D


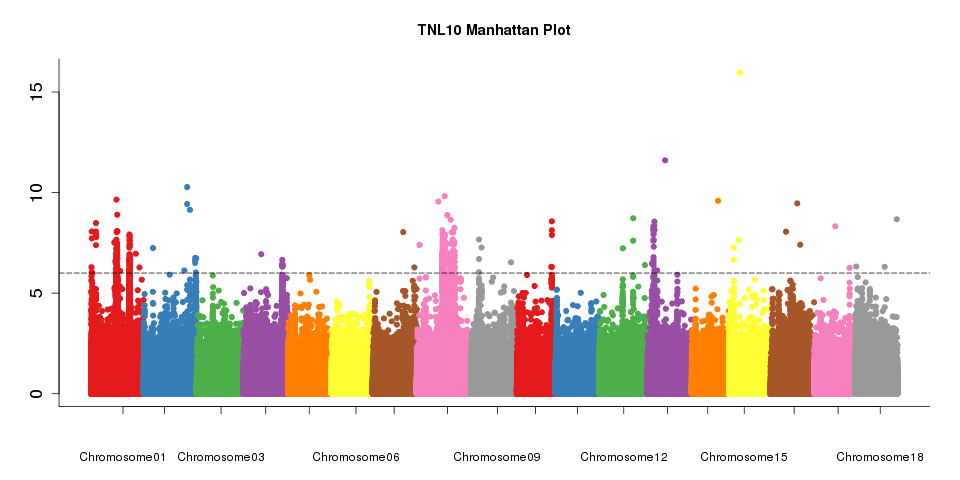


E


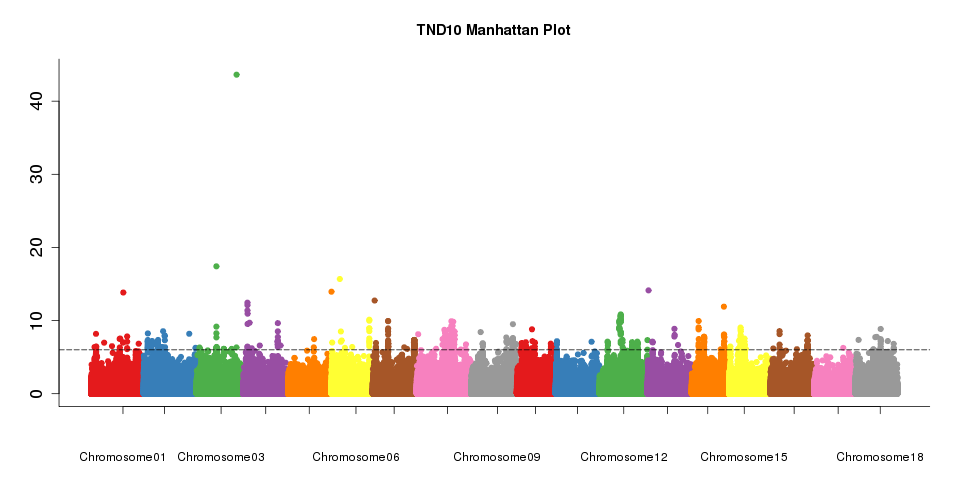


**Supplementary Figure S3 | Manhattan plot of five traits associated to low-temperature stress in 170 cassava germplasms under low-temperature stress.**

1. Plant height after low temperature (cm) PHA
2. Total Number of Leaves after low temperature TNL
3. Total Number of damaged Leaves after low temperature TND
4. Total Number of leaves grown in the first 10 internodes after low temperature TNL10
5. Total Number of damaged leaves in the first 10 internodes after low temperature TND10

Total Number of Leaves after low temperature TNL for further analyzed in this study.

**Supplementary Table S1 | Summary statistics of low temperature tolerance phenotypes in low temperature stress experiments.**

| Traits | Organ | Treatment  /Control | Minimum | Maximum | Average | SD | CV(%) | ANOVA  F value p value |
| --- | --- | --- | --- | --- | --- | --- | --- | --- |
| POD | leaf | Control | 98.55 | 64413 | 8100 | 12366 | 152.7 | 14.84 1.0E-4 |
|  |  | Treatment | 85.43 | 84236 | 11127 | 18124 | 162.90 |  |
|  | root | Control | 91.22 | 66649 | 10423 | 15180 | 145.60 | 0.8937 0.6476 |
|  |  | Treatment | 83.77 | 78287 | 12134 | 18076 | 149.00 |  |
| CAT | leaf | Control | 2.3 | 961.9 | 152.9 | 201.6 | 131.80 | 0.7874 0.7584 |
|  |  | Treatment | 1.9 | 894.7 | 149.9 | 193.1 | 128.80 |  |
|  | root | Control | 2.5 | 1050 | 116 | 220.4 | 190.0 | 24.19 2.0E-4 |
|  |  | Treatment | 1.90 | 989.3 | 94.93 | 203.0 | 213.8 |  |
| SOD | leaf | Control | 22.55 | 445.2 | 173.8 | 87.68 | 50.46 | 2.545 2.2E-3 |
|  |  | Treatment | 22 | 482.7 | 181.7 | 119.3 | 65.88 |  |
|  | root | Control | 31 | 468.2 | 161.1 | 85.57 | 53.10 | 1.191 0.2937 |
|  |  | Treatment | 22.33 | 475.9 | 168.7 | 93.8 | 55.61 |  |

| **Supplementary Table S2 \| Correlation analyses among MebHLH18 expression, leaf abscission rate, and POD activity.** | | | | | | | | |
| --- | --- | --- | --- | --- | --- | --- | --- | --- |
| MebHLH18 expression under low temperature stress | | | | |  |  |  | r |
| 0 | 6h | 12h | 24h | 48h | 72h |  |  |  |
| 1.1 | 2.3 | 3.2 | 4.5 | 5.3 | 7.1 |  |  |  |
| 1 | 2.2 | 2.8 | 4.1 | 5.9 | 5.9 |  |  |  |
| 1.11 | 1.5 | 2.9 | 4.6 | 4.8 | 6.7 |  |  |  |
|  |  |  |  |  |  |  |  |  |
| Leaf abscission rate at 10 days recovery growth after low temperature treatment | | | | | | | |  |
| 0 | 6h | 12h | 24h | 48h | 72h |  |  | 0.967349 |
| 0 | 0 | 13 | 45 | 51 | 78 |  |  |  |
| 0 | 6 | 5 | 50 | 59 | 69 |  |  |  |
| 0 | 0 | 8 | 43 | 47 | 81 |  |  |  |
|  |  |  |  |  |  |  |  |  |
| POD activiy under low temperature stress | | | |  |  |  |  |  |
| 0 | 6h | 12h | 24h | 48h | 72h |  |  | 1 |
| 1.1 | 2.3 | 3.2 | 4.5 | 5.3 | 7.1 |  |  |  |
| 1 | 2.2 | 2.8 | 4.1 | 5.9 | 5.9 |  |  |  |
| 1.11 | 1.5 | 2.9 | 4.6 | 4.8 | 6.7 |  |  |  |

**Supplementary Table S3 | Selected 170 cassava germplasms and the TNL value.**

| Cassava germplasm | TNL | Cassava germplasm | TNL | Cassava germplasm | TNL |
| --- | --- | --- | --- | --- | --- |
| SwissNO12 | 21.75 | Wenchanghongxi | 11 | Ba-1 | 77.33333 |
| Ecu83 | 24.67 | Ecu84 | 13 | 1-3 | 22.6667 |
| CM92-56-1 | 9.5 | Brazil 9 | 19.25 | 2-73 | 43.33333 |
| No. 1 Guizhou | 54 | 033 | 22 | SC6 | 22.75 |
| Thai species | 27 | FLAxxx-12 | 47.33 | 037 | 15.5 |
| 287 | 70.5 | ZM7901 | 46.25 | 035 | 21 |
| 13C004 | 20.33333 | Swiss88 | 13 | BRA-V | 87 |
| 2-18 | 11.25 | 281 | 22 | BRA646 | 42 |
| CM7595-1 II183 | 20.5 | Brazil 10 | 48.66667 | SC124 polyploid | 26 |
| R7 | 22.33 | GR024-2 | 0.33 | SC8013 | 41 |
| 13C007 | 4.666667 | SC14 | 13.5 | Hanate | 40.75 |
| ZM9066 | 27.66667 | 021 | 32.33333 | Nanzhi188 | 47.25 |
| SSA19 | 24 | ZMD578 | 192.33 | 2-41 | 4.333333 |
| COL777 | 30 | SC13 | 56.66667 | Baodao9-3 | 3 |
| CMR35-22-196 | 65.66667 | Baodao9-5 | 14.25 | Laos Banrajau | 7.75 |
| 2-60 | 74 | ZMF642 | 65.33333 | 2-20 | 49 |
| Guangximushu | 2.33 | 2-30 | 38.33333 | Yunnan No. 8 | 26.75 |
| 310 | 28.66667 | 3555-6 | 11 | SwissS8 | 14.66667 |
| SC6068 | 25.88 | 237 | 44 | 260 | 62.66667 |
| 60 | 31.5 | CM385-6 | 7 | 010 | 1.333333 |
| ZMC635 | 64.33 | 2-85 | 128 | 028 | 23.75 |
| 027 | 4.333333 | 034 | 17.66667 | Zhedongzhenjie | 61.75 |
| Guire no. 4 | 24.66667 | CM901 | 36.33 | Baodao9-1 | 20.5 |
| ZMC1115 | 17.25 | 2-61 | 14.8 | FLAxxx-25 | 10.5 |
| 2-51 | 2.75 | Indonesian foliage | 35 | BX-2 | 16.66667 |
| GR024-8 | 42.75 | Tai Yin No. 1 | 182.67 | CM965-3 | 44.67 |
| 332 | 99 | CMR37-14-9 | 13 | 005 | 3.666667 |
| 2-74 | 56 | COL629-4 | 1.5 | CM7595-1 II178 | 0 |
| SwissD23 | 5.666667 | Laotian shike | 30.75 | 2-4 | 79 |
| Zhihui | 23 | CMR36-40-9 | 8.67 | 2-78 | 59 |
| 184 | 57 | Malay 5 | 96 | ZM9242 | 17 |
| 270 | 15.5 | SwissR9 | 49.33333 | Liming | 28 |
| SC8002 | 24.67 | SCT104-264 | 0.5 | COL198 | 48 |
| COL2626 | 57.33 | 4 | 43.66667 | SwissX3 | 51.67 |
| Indonesian species | 65 | COL588 | 153 | KU50 | 37.25 |
| 19 | 54.66667 | ZMF821 | 45 | ZM93236 | 39 |
| CM1568-2 | 0 | 103 | 19 | 2-28 | 2.5 |
| A8 | 75.66667 | BX-1 | 15 | 023 | 6 |
| GR024-9 | 52.33333 | M.COL22 | 22 | R60 | 24 |
| SC9 | 26.67 | SC124 | 43 | 14-53 | 33 |
| BRA274 | 8 | 13C005 | 110.6667 | 003 | 27.33333 |
| ZM8229 | 59.25 | IB05 | 67.66667 | 2-49 | 86.66667 |
| ZM99247 | 40.33 | Gui Re No. 5 | 61 | 020 | 24 |
| SC5 | 112.1667 | New Election 048 | 5.67 | 2-19 | 41 |
| 2-53 | 38.5 | 14-43 | 75.5 | I93 | 34.33333 |
| 171 | 20.5 | 024 | 20 | SwissT7 | 34.66667 |
| Fujian huaan | 13 | 009 | 41 | 032 | 41.5 |
| SwissU6 | 40.33 | 14 | 1 | Brazil 14 | 30.33333 |
| Andera | 72.5 | 019 | 32 | SC205 | 12 |
| 369 | 33.5 | 242 | 30.5 | 9006 | 56 |
| 2-27 | 41 | COL1175 | 77.66667 | 029 | 14.66667 |
| 44-1-1 | 49.33333 | Qiongzhong No 1 | 50.66667 | 017 Brown | 7 |
| 2-21 | 36.33333 | COL784 | 30 | ZM99250 | 29.5 |
| 001 | 67.5 | 026 | 32.25 | 2-71 | 38 |
| Rubber cassava | 76.5 | SM2300-1 | 131.3333 | CM3993-9 | 17.33 |
| 13C008 | 70 | 006 | 8.5 | 016 | 3 |
| M.COL1468 | 42 | COL514 | 0 |  |  |

TNL: Take every 5 plants as a sample, repeat the sampling for three times, and take the average value.

**Supplementary Table S4 | Five traits were measured in 170 cassava germplasms under low-temperature stress.**

1. Plant height after low temperature (cm) PHA
2. Total Number of Leaves after low temperature TNL
3. Total Number of damaged Leaves after low temperature TND
4. Total Number of leaves grown in the first 10 internodes after low temperature TNL10
5. Total Number of damaged leaves in the first 10 internodes after low temperature TND10

**Supplementary Table S6 | The list of primers used in this study.**

| Primers for real-time PCR | |
| --- | --- |
| bHLH18-RT-F | TCATGAGACCAATAGCTGGAAC |
| bHLH18-RT-R | GTTCTGGTCATCGAGTAAG |
| ACT7-F | ACAGAGAGAAGATGACCCAAATC |
| ACT7-R | CCATCACCAGAATCCAGTACAA |
|  |  |
| Primers for gene transformation vector construct | |
| OE-F | agtggtctctgtccagtcctATGGATATAGCTCCGGCGAGAT |
| OE-R | ggtctcagcagaccacaagtCTACATGAACTTCAAAAAAGCCAC |
| RNAi-F | agtggtctctgtccagtcctTCATGAGACCAATAGCTGGAAC |
| RNAi-R | ggtctcagcagaccacaagtGTTCTGGTCATCGAGTAAG |
|  |  |
| Primers for MebHLH18 Promoter | |
| P-F | GGCAAGCAAGTCGGTGAAG |
| P-R | TACGGCAGCTTGTATTTTCAAGG |
|  |  |
| Primers for MebHLH18 Promoter mutation | |
| MP-F1 | GCATTTGAATAGTTGCAGAAGATGAAGACAAATTTTCACACGTGGGT |
| MP-R1 | ACCCACGTGTGAAAATTTGTCTTCATCTTCTGCAACTATTCAAATGC |
| MP-F | agtggtctctgtccagtcctCTGCATTTGAATAGTTGCAGAAGAT |
| MP-R | ggtctcagcagaccacaagtTACGGCAGCTTGTATTTTCAAGG |
|  |  |
| Primer for MebHLH18 for nucleus-localization analysis | |
| OE-F | agtggtctctgtccagtcctATGGATATAGCTCCGGCGAGAT |
| OE-R | ggtctcagcagaccacaagtCTACATGAACTTCAAAAAAGCCAC |
